# Fresh takes on five health data sharing domains: Quality, privacy, equity, incentives, and sustainability

**DOI:** 10.3389/fdata.2023.1095119

**Published:** 2023-02-06

**Authors:** Christi J. Guerrini, Mary A. Majumder, Jill O. Robinson, Robert Cook-Deegan, Matthew Blank, Juli Bollinger, Janis Geary, Amanda M. Gutierrez, Maya Shrikant, Amy L. McGuire

**Affiliations:** ^1^Center for Medical Ethics and Health Policy, Baylor College of Medicine, Houston, TX, United States; ^2^Consortium for Science, Policy & Outcomes, Arizona State University, Washington, DC, United States

**Keywords:** data sharing, data quality, ethics, data privacy, data archives

## Abstract

As entities around the world invest in repositories and other infrastructure to facilitate health data sharing, scalable solutions to data sharing challenges are needed. We conducted semi-structured interviews with 24 experts to explore views on potential issues and policy options related to health data sharing. In this Perspective, we describe and contextualize unconventional insights shared by our interviewees relevant to issues in five domains: data quality, privacy, equity, incentives, and sustainability. These insights question a focus on granular quality metrics for gatekeeping; challenge enthusiasm for maximalist risk disclosure practices; call attention to power dynamics that potentially compromise the patient's voice; encourage faith in the sharing proclivities of new generations of scientists; and endorse accounting for personal disposition in the selection of long-term partners. We consider the merits of each insight with the broad goal of encouraging creative thinking to address data sharing challenges.

## Introduction

It is widely agreed that sharing health data will translate to benefits for patients and populations and is critical to the advancement of science (Institute of Medicine of the National Academies, 2013; Editorial, 2020; Whicher et al., 2021). The widely cited and endorsed FAIR Guiding Principles provide an invaluable foundation for data management and stewardship (Wilkinson et al., 2016). However, technical, motivational, and policy barriers to sharing health data for secondary research persist [National Academies of Sciences, Engineering, and Medicine (NASEM), 2018]. As public and private entities increase investments in repositories and other infrastructure to facilitate health data sharing, scalable approaches to overcoming these barriers are urgently needed (Institute of Medicine of the National Academies, 2013; Whicher et al., 2021).

Addressing this need, we conducted a modified policy Delphi process to identify and prioritize issues and policy options related to sharing cancer-gene variant data (Majumder et al., 2021). Cancer genomics was the focus of our research given the field's significant efforts to make large-scale data sets available for secondary research with the objective of, among other things, resolving problems concerning variants of uncertain significance (The Clinical Cancer Genome Task Team of the Global Alliance for Genomics and Health, 2017). In the first three Delphi rounds, panelists prioritized issues and generated potential options that we categorized into five domains: data quality, privacy and security, equity, incentives, and sustainability. To broaden the range of perspectives considered by panelists in the final Delphi round, we conducted semi-structured interviews with 24 experts who did not participate in the Delphi process (Table 1). Methods for recruiting interviewees, conducting interviews, and analyzing interview data are described in Supplementary material.

**Table 1:** Characteristics of interview participants (*N* = 24).

	***n* (%)**
**Gender**	
Male	11 (46)
Female	13 (54)
Prefer to self-describe	–
**Age, in years**	
35–45	7 (29)
46–55	4 (17)
56–65	4 (17)
66–75	2 (8)
Missing data^*^	7 (29)
**Residence**	
U.S.	20 (83)
Non-U.S.	3 (13)
Missing data^*^	1 (4)
**Role(s) relevant to health data sharing** ^**^	
Data contributors/end-users	4 (17)
Data generators	1 (4)
Data sources	3 (13)
Data facilitators	1 (4)
Professional data users	12 (50)
Policy experts/scholars	10 (42)
Other	3 (13)
Missing data^*^	5 (21)

* Response was not forced.

** Interviewees were asked to select their role(s) related to cancer genomics commons from the following options: data contributors/end-users=patients, families, and advocacy organizations; data generators=testing laboratories; data sources=databases; data facilitators=data resources, curators, annotators, and variant interpreters; professional data users=genetic counselors, clinicians, and researchers; policy experts/scholars=health and biomedical research policy experts and scholars. Options were select all that apply so total exceeds *N* = 24 (100%).

In this Perspective, we describe and contextualize select insights of interviewees on data sharing that we found intriguing and generated rich discussion among our research team (Table 2). Importantly, these insights are not limited to cancer genomics but are relevant to any efforts to share health data. We do not claim that these perspectives have never before been aired, but because they depart (in some cases significantly) from conventional thinking, we refer to them as “fresh takes.” Although some might be controversial, we believe each has sufficient merit to justify exploration. More generally, by airing these fresh takes, we aim to encourage consideration of novel approaches to sharing health data.

**Table 2:** Insights on health data sharing issues shared by interviewees (*N* = 24).

Issue domain^*^	**Conventional approach**	**Fresh take**	Supporting quote from interviews^**^
Data quality	Standards should be developed for judging data as high or low quality with the goal of generating, sharing, and reusing primarily high-quality data.	Standards should be developed for characterizing data with the goal of understanding why and how they were generated and what they do and do not describe. Appropriateness of reuse depends on context—specifically, alignment of data characteristics with the objectives and needs of specific studies.	“[W]e should be trying to move away from creating data standards for every data point.... [W]e've been trying to do that for two decades and it really has been a fair amount of nonsense. But rather, there should be a very consistent way of describing and characterizing data quality or characterizing data.... I think that quality ascribes judgement, and all data have warts.” (9)
Privacy	In relation to research participants and the general public, data holders and managers should be transparent about privacy and security risks because doing so demonstrates respect and helps build trust. More disclosure is generally better than less disclosure.	Emphasizing privacy risks and security protections can breed mistrust. Policy attention should focus on promoting an appropriate level of disclosure, which should take into account psychological impacts.	“[I]f you want to build trust... don't speak about privacy too much.... [I]t goes back to the psychology... Oh, do you want this coffee? This coffee is without poison. And then it's like, Wait, of course, why do you tell me that it's without poison?” (10)
Equity	Patients and their representatives should be included in data governance to help ensure that decisions are responsive to their interests and concerns.	If patients and their representatives are to fulfill the role envisioned for them, power dynamics inherent in decision making processes must be recognized and managed. More broadly, equity initiatives should recognize epistemic equity as an area requiring attention and action.	Patients and their representatives invited to meetings with research funders can be told, “Don't say this or don't say that.” Funders might focus on, “We're including a patient advocate, we're having the patient's voice at the table” and not know that the “patient's voice has already been filtered, is already being dominated, if you will.” (21)
Incentives	Researchers can be reluctant to share data because doing so is not always in their professional interests. Policy efforts should therefore focus on creating incentives and removing disincentives for sharing.	The culture of newer generations of researchers is to share data. It is unnecessary to devote significant policy attention to incentives and disincentives because this culture will eventually be dominant.	If “you look at people that had got their PhDs within the last 10 years, they're probably much more active in the open science community.... And so, I actually see this as a problem that's going to be taken care of by the natural course of familiarity with a new way of working, which is digital, and that it's correcting itself. And if you say, how do you accelerate it?..... I'd probably answer back: is it worth trying to accelerate, or is it worth just promoting, helping those people that are operating in the new model be successful?” (23)
Sustainability	Partners should be recruited based on expertise, prestige, and resources.	All partners should be critically assessed to ensure that personal dispositions will promote rather than hinder the long-term success of health data repositories for sharing.	It's important to maintain “a pretty hard line on keeping the assholes out.... [T]here are some people who are poison to any consortium and you just can't have them involved....” (8)

* Issue domains identified during modified policy Delphi process (Majumder et al., 2021).

** Interviewee designated by number in parentheses.

## Data quality: Questioning a focus on granular quality metrics for gatekeeping

The first fresh take focuses on the consequences of sharing data judged to be low quality. The conventional approach is to develop standards by which to designate data as high or low quality with the goal of generating, sharing, and reusing primarily high-quality data. One interviewee, however, worried about generalized use of metrics to expunge or block data from repositories, based on a judgment that they are low-quality according to those metrics, because “all data have warts.” Depending on the specific objectives and needs of studies that might reuse data, the interviewee suggested, a data set's particular blemishes might not be significant or even relevant. To help researchers make decisions about reusing data, standards should therefore be developed for characterizing why and how the data were generated, and what they do and do not describe, to promote understanding of their strengths and limitations for specific secondary use contexts.

Consistent with the notion of quality as fitness for use, information systems professionals have described quality dimensions from the perspective of data users that include extrinsic indicators of contextual appropriateness, such as relevance to the task at hand and completeness, in addition to intrinsic indicators, such as accuracy (Wang and Strong, 1996). Medical researchers also recognize that annotation of data facilitates reuse, but data quality frameworks generally focus on development of and compliance with quality standards or metrics. In a systematic review of frameworks for data sharing within consortium-wide platforms for international health research, for example, principles and norms for data sharing included development and implementation of quality standards or threshold metrics (Kalkman et al., 2019). Our interviewee's unconventional insight is that the “play books” of primary researchers—e.g., the rich, narrative descriptions of how the data were originally generated, coded, and interrogated (Bauchner et al., 2016)—are as or even more useful to secondary researchers than granular quality metrics, especially those focused on identifying and quantifying quality “defects” as a basis for exclusion from the data commons. More broadly, it is worth considering whether use of the term “quality” promotes simplistic judgements about data that discourage appropriate reuse.

## Privacy: Challenging maximalist disclosures about data sharing risks

The second fresh take concerns research participants' privacy and challenges with keeping their data confidential once shared. Because it is usually impossible to guarantee that data will never come into the possession of unauthorized persons or be used for unauthorized purposes, including reidentification, the conventional wisdom is that disclosing more information about privacy risks is generally better than less. Transparency is also believed to promote trust. One of our interviewees, however, argued that privacy-related disclosures can have the opposite effect by arousing suspicion. By analogy, the interviewee described a neighborhood coffee shop that assures customers its coffee is poison-free. Because customers do not normally wonder whether their coffee is laced with arsenic, the assurance causes customers to worry and ask, “Wait... why do you tell me that it's without poison?” The interviewee therefore advised, “if you want to build trust... don't speak about privacy too much.”

A recent study suggests that members of the public who are open to donating their health data for research believe that transparency about how their data are used would help them trust the data-sharing enterprise (Milne et al., 2021). However, people's views and behaviors around privacy and related trade-offs are more uncertain, malleable, and context-dependent than is often recognized (Acquisti et al., 2015). Indeed, limited attention, “motivated attention” away from unpleasant information, and biased assessments of probability can diminish or even reverse intended effects of privacy-related disclosures (Loewenstein et al., 2014). Further, groups can have different levels of pre-existing concern about privacy that influence how disclosures affect trust. Still, some advocates for disclosure may appeal to considerations such as respect as justification for transparency regardless of any effects on trust (McGuire et al., 2019). In sum, many factors complicate the relationship between disclosures and their impact on trust and accountability (Loewenstein et al., 2014). The nugget of wisdom here is to be curious about and account for human psychology when obtaining consent for sharing health data and beware of unreflective disclosure maximalism.

## Equity: Calling attention to power dynamics that potentially compromise the patient's voice

The third fresh take is in the domain of equity. Increasing diversity and sensitivity to the needs and concerns of patients and communities have recently been articulated as priorities in biomedical research (Aguilar-Gaxiola et al., 2022). Consistent with these priorities, some have championed biobank and data repository systems and processes that engage the general public, patients, and patient representatives in data governance (O'Doherty et al., 2011; Kaye et al., 2018; McGuire et al., 2019). But one of our interviewees cautioned that “having the patient's voice at the table” is not in itself sufficient to achieve equity. This is due to inherent power differences between patients and researchers—who may also be treating physicians. We might expect those desperate for help to avoid doing anything that might alienate those researchers. Thus, the interviewee observed, patients might not use their authentic voices—and might even simply parrot what researchers tell them to say—when invited to the table.

The interviewee's concerns are relevant to what Miranda Fricker calls epistemic injustice (Fricker, 2003). Epistemic injustice can occur when a hearer (e.g., physician or researcher) assigns lower credibility to a speaker (e.g., patient or caregiver) as a result of a prejudice stemming from differences of social identity, especially where the differences are characterized by unequal power between the hearer and the speaker (Fricker, 2003). It can also occur preemptively when the speaker remains silent out of fear of not being believed (Lee, 2021). Our interviewee's novel insight is that such silencing can occur out of fear of disrupting existing relationships *as a result* of being believed. Therefore, to protect against the (sometimes unintentional) filtering or dominance of the patient's voice, upstream solutions are needed to better identify and manage relevant power dynamics. For example, data governance can be structured to require the input of many patients and caregivers, rather than just a few.

## Incentives: Focusing efforts on new generations of scientists

The fourth fresh take addresses the misalignment of data sharing with researchers' professional incentives. The conventional approach to this well-known problem is to reward data sharing, reduce professional incentives for data hoarding, and enshrine data sharing as an institutional and cultural norm. There are many examples of efforts that have adopted this approach, including the use of sharing badges by journals and data advertising by consortia to enhance the visibility of data sets and reputational credit of their creators (Devriendt et al., 2021). One of our interviewees, however, wondered whether these approaches are necessary given the popularity of open science norms among scientists who have pursued advanced degrees “within the last 10 years.” They explained: “I actually see this as a problem that's going to be taken care of by the natural course of familiarity with a new way of working, which is digital, and that it's correcting itself.” To those asking how to accelerate this change, the interviewee continued, “I'd probably answer back: is it worth trying to accelerate, or is it worth just promoting, helping those people that are operating in the new model be successful?”

Because incentive-related barriers to health data sharing have proven especially tricky to overcome, the wait-it-out approach endorsed by this interviewee has undeniable appeal. It is also true that there is broad enthusiasm for open science, as evidenced by global initiatives to facilitate access to research data, methods, and products [National Academies of Sciences, Engineering, and Medicine (NASEM), 2018]. Yet, one survey of over 1,300 scientists found that, compared to their older colleagues, younger scientists were less willing to share their research data without restriction, although they were more likely to agree that lack of access to data is a major impediment to progress in science and has restricted their ability to answer scientific questions (Tenopir et al., 2011). Other scholars attribute this finding to competitive pressures that are likely experienced more intensely by non-tenured scientists compared to their tenured colleagues (Fecher et al., 2015). More generally, the literature suggests that incentives and norms should move in the same direction to sustain behavior (Nicholas et al., 2019), and so it does not seem wise to disinvest in incentives for data sharing. Still, the interviewee's insight is useful in thinking about how to maximize the impact of those investments: instead of working to change the behaviors of a resistant old guard, focus on supporting new generations of scientists who might be more receptive to sharing.

## Sustainability: Endorsing personal disposition as a partner screen

The final fresh take concerns the financial and human resource challenges associated with maintaining data repositories and sharing programs. A standard approach to promoting sustainability is to partner with individuals and institutions based on their access to resources, as well as their expertise and prestige, which can help attract external funding. But one of our interviewees recommended including an additional screen for personal disposition. Specifically, they explained, it is important to maintain “a pretty hard line on keeping the assholes out.” The interviewee elaborated: “[T]here are some people who are poison to any consortium and you just can't have them involved.”

Following publication of Sutton's (2004, 2007) landmark *Harvard Business Review* essay in 2004 and follow-on book in 2007, the “no asshole rule” has become well-known in management circles. This rule is intended to protect organizational culture by denying entry (usually in the form of employment) to even high-achieving individuals if they are known to exhibit abusive or other difficult behavior. The interviewee's novel insight was recognizing its relevance beyond business hiring contexts and applying it to decisions about partners in long-term and large-scale scientific collaborations. The move provokes a broader question: what other lessons about long-term operational success might data sharing efforts glean from the business management literature?

## Conclusion

Given the intractability of issues associated with developing and sustaining repositories and other infrastructure to facilitate health data sharing, we believe it is worth paying attention to these and other unconventional perspectives. They have the potential to generate new and better solutions by drawing from literature in different fields, highlighting edge and hidden cases, and even reframing the problems. While not every fresh take will ultimately be useful to efforts to promote health data sharing, soliciting and airing them can help ensure that this work is conducted in ways that are open-minded and creative.

## Data availability statement

The data presented in this article are not readily available due to the confidential nature of interviewees' participation and consistent with the IRB-approved protocol for this study. Requests to access the data should be directed to guerrini@bcm.edu.

## Ethics statement

The studies involving human participants were reviewed and approved by Baylor College of Medicine Institutional Review Board. Written informed consent for participation was not required for this study in accordance with the national legislation and the institutional requirements.

## Author contributions

AM, CG, MM, and JR conceived of and designed this study. CG, MM, RC-D, JB, JG, and AG participated in data collection with support provided by JR and MB. CG, MM, JR, MB, and MS analyzed the data. CG, MM, and JR led the drafting of this manuscript. Funding for this research was obtained by RC-D and AM. All authors contributed to the article and approved the submitted version.
