# Supplementary material for: Fresh takes on five health data sharing domains: Quality, privacy, equity, incentives, and sustainability
Source: Front Big Data. 2023 Feb 6;6:1095119. doi: 10.3389/fdata.2023.1095119 (PMC9939819; doi:10.3389/fdata.2023.1095119)
Supplement: Supplementary file 1 [file Data_Sheet_1.pdf]

Supplementary Material

Methods

From February to November 2021, we conducted semi-structured interviews with 24 individuals as part of the Sulston Project (R01 CA237118), which is examining issues and potential policy options related to sharing cancer gene variant data. To be considered for inclusion in the interview study, each candidate was required to be at least 18 years old; be able to participate in an English-language interview; and have worked to address challenges of health data sharing relevant to one or more of five domains, as evidenced by professional activities, presentations, or publications. The five domains were generated from early rounds of a modified policy Delphi process that is also part of the Sulston Project (Majumder et al., 2021). Demographic factors of interview candidates were also taken into consideration to enhance the diversity of opinions.

Each candidate was contacted via email with an invitation to participate that described the purpose of the study. If the candidate did not respond to the invitation, up to two follow-up emails were sent. No additional emails were sent after the second follow-up email. We contacted 35 candidates in total. Three declined and four did not respond, for a response rate of 80%. Four of the 28 candidates who agreed to participate ultimately were not scheduled for an interview because the study team concluded that thematic saturation had been reached.

Interviews were conducted using a semi-structured interview guide. Interviews were conducted by Zoom and audio recorded with permission. Each interviewee provided verbal consent to participate at the beginning of the interview and was offered a $50 gift card for their participation. Interviews ranged from 54 to 67 minutes; the mean interview length was 58 minutes.

At the conclusion of each interview, the audio recording was professionally transcribed and the transcript checked for fidelity to the audio recording and deidentified. Clean and deidentified transcripts were entered into Dedoose Version 9.0.54, a web-based application for managing and storing qualitative data (2019, Los Angeles, CA: SocioCultural Research Consultants, LLC).

Transcripts were coded for nuances to policy options identified earlier in the Delphi process; new policy options; and fresh takes. In the first round of coding, two authors coded all transcripts. In the second round, four authors reviewed the coded transcripts for coding accuracy and consistency. As a result of application of this process, for each transcript, the assignment of codes to the transcript was evaluated by some combination of two authors; any disagreement was resolved by those two authors. The coded transcripts were then reviewed by the project team to identify a range of policy options relevant to health data sharing, including unconventional approaches.

This research was approved by the Baylor College of Medicine Institutional Review Board.

References:

Majumder, M. A., Blank, M. L., Geary, J., Bollinger, J. M., Guerrini, C. J., Robinson, J. O., et al. (2021). Challenges to building a gene variant commons to assess hereditary cancer risk: results of a modified policy Delphi panel deliberation. *J. Pers. Med.* 11, 646.
